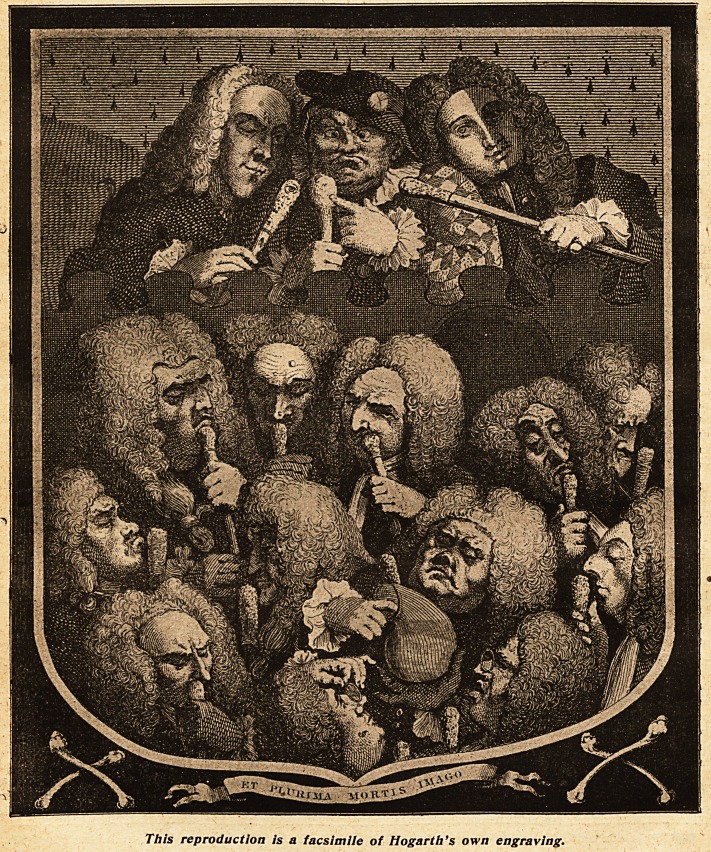# Designed by Hogarth

**Published:** 1918-06-22

**Authors:** 


					A CONSULTATION OF PHYSICIANS.
DESIGNED BY HOGARTH.
mm
Fv. ,
>{?
E t
?
r VI A AI<)1VV1^
.vl?
T/j/s reproduction is a facsimile of Hogarth's own engraving.

				

## Figures and Tables

**Figure f1:**